# Executive function during exercise is diminished by prolonged cognitive effort in men

**DOI:** 10.1038/s41598-022-26788-6

**Published:** 2022-12-27

**Authors:** Hayato Tsukamoto, Kento Dora, Benjamin S. Stacey, Hibiki Tsumura, Yoshino Murakami, Christopher J. Marley, Damian M. Bailey, Takeshi Hashimoto

**Affiliations:** 1 Faculty of Sport and Health Science, Ritsumeikan University, 1-1-1 Nojihigashi, Kusatsu, Shiga 525-8577 Japan; 2 Neurovascular Research Laboratory, Faculty of Life Sciences and Education, University of South Wales, Pontypridd, UK; 3 Department of Biomedical Engineering, Toyo University, Kawagoe, Saitama Japan

**Keywords:** Neuroscience, Physiology, Psychology

## Abstract

The speed and accuracy of decision-making (*i.e.*, executive function (EF) domains) is an integral factor in many sports. At rest, prolonged cognitive load (pCL) impairs reaction time (RT). In contrast, exercise improves RT and EF. We hypothesized that RT and EF during exercise would be diminished by prolonged ‘dual tasking’ as a consequence of pCL. To test the hypothesis, twenty healthy male participants performed four conditions [resting control (Rest), pCL only (pCL_Rest_), exercise only (EX), and pCL + exercise (pCL_EX_)] in a randomized-crossover design. Both exercise conditions utilized a 50-min cycling exercise protocol (60% VO_2_ peak) and the pCL was achieved via a 50-min colour-word Stroop task (CWST). Compared with Rest, pCL_Rest_ caused a slowed CWST RT (*P* < 0.05) and a large SD (*i.e.*, intraindividual variability) of CWST RT (*P* < 0.01). Similarly, compared with EX, the slowed CWST RT (*P* < 0.05) and large SD of CWST RT (*P* < 0.01) were also observed in pCL_EX_. Whereas the reverse-Stroop interference was not affected in pCL_Rest_ (*P* = 0.46), it was larger (*i.e.*, declined EF) in pCL_EX_ than EX condition (*P* < 0.05). These observations provide evidence that the effort of pCL impairs RT and EF even during exercise.

## Introduction

Well-developed cognitive abilities (*e.g.*, the speed and accuracy of decision-making) are an integral factor contributing to enhanced sporting performance and are thought to be as important as technical skills (*e.g.*, ball control with a body), athlete physique (*e.g.*, height and weight) and physiological components (*e.g.*, strength and cardiorespiratory fitness)^1,2^. Reilly^3^ speculated that the performance in sports such as football may be gradually impaired towards the end of the game partly due to mental fatigue. Given that mental fatigue negatively influences cognitive performance^4,5^, it seems reasonable to hypothesize that the speed and accuracy of decision-making are impaired with accumulating levels of mental fatigue (*e.g.*, in the latter stages of the match)^6–8^.

The speed and accuracy of decision-making are controlled by executive function (EF)^9^, which involves three aspects: shifting of mental sets, monitoring and updating of working memory representations, and inhibition of prepotent responses^10^. It is well-established that moderate-intensity aerobic exercise increases state mental fatigue but acutely improves cognition such as EF^11–16^. However, cognitive decline can be observed during prolonged exercise when brain activation (*e.g.*, motor area) gradually approaches the limited capacity of information processing due to extremely increased ‘fatigue’ (*e.g.*, peripheral and mental exhaustion etc.)^17^. Given that state mental fatigue may be increased by not only aerobic exercise effort^13,14^ but also cognitive effort^18,19^, the time-to-mental exhaustion may be reached faster during the combination of aerobic exercise and cognitive effort (*i.e.*, a dual-task with motor and cognitive demands) than aerobic exercise only (*i.e.*, a single-task with motor demands). Namely, it could be suggested that state mental fatigue is further augmented by prolonged multiple-task performance, which is continuously carried out during sports (*e.g.*, decision-making while running).

Completing a single cognitive task at rest (> 30 min) has the capacity to impair cognitive-perceptive skills^4,19,20^. For instance, the effort in prolonged cognitive load (pCL) induced by the colour-word Stroop task (CWST) which can assess inhibitory control in EF causes slowed reaction times (RT) and large intraindividual RT variability along with mental fatigue (called cognitive fatigue)^4^. In addition to RT, cognitive fatigue impairs sport-related physical performance such as time-to-exhaustion^19^. In the real sports setting, a recent study demonstrated that pCL-induced cognitive fatigue prior to competition not only impairs aspects of cognitive and tactical performance, but results in a compensatory increase in physical demands for athletes (*e.g.,* running distance)^20^. However, it is unclear to what extent the pCL impairs cognitive-perceptive abilities during moderate-intensity aerobic exercise. In the present study, we hypothesized that mental fatigue in response to a 50-min moderate-intensity aerobic exercise with pCL would be higher than aerobic exercise only. In this context, we also hypothesized that a 50-min pCL would slow RT during acute moderate-intensity aerobic exercise, even though our previous study demonstrated that the speed of decision-making is gradually shortened during 50-min moderate-intensity aerobic exercise^12^. To test our hypotheses, the aim of the present study was to examine the impact pCL during acute exercise has on RT and the accuracy of the EF task (*i.e.*, CWST) in addition to the psychological state.

## Results

Due to volitional exhaustion, one participant could not complete the exercise protocol for 50-min and was therefore excluded. Data from the remaining 20 participants (means ± SD; age 22 ± 2 years, height 173 ± 4 cm, weight 64 ± 5 kg, peak oxygen uptake (VO_2_ peak) 45.6 ± 5.0 ml/kg/min) were used for analysis. There were no significant differences in parameters at baseline between conditions (1, resting control without pCL (Rest); 2, rest with pCL (pCL_Rest_); 3, exercise without pCL (EX); and 4, exercise with pCL (pCL_EX_)) (Supplementary Table 1).

### Psychological responses and heart rate

**Figure 1:**
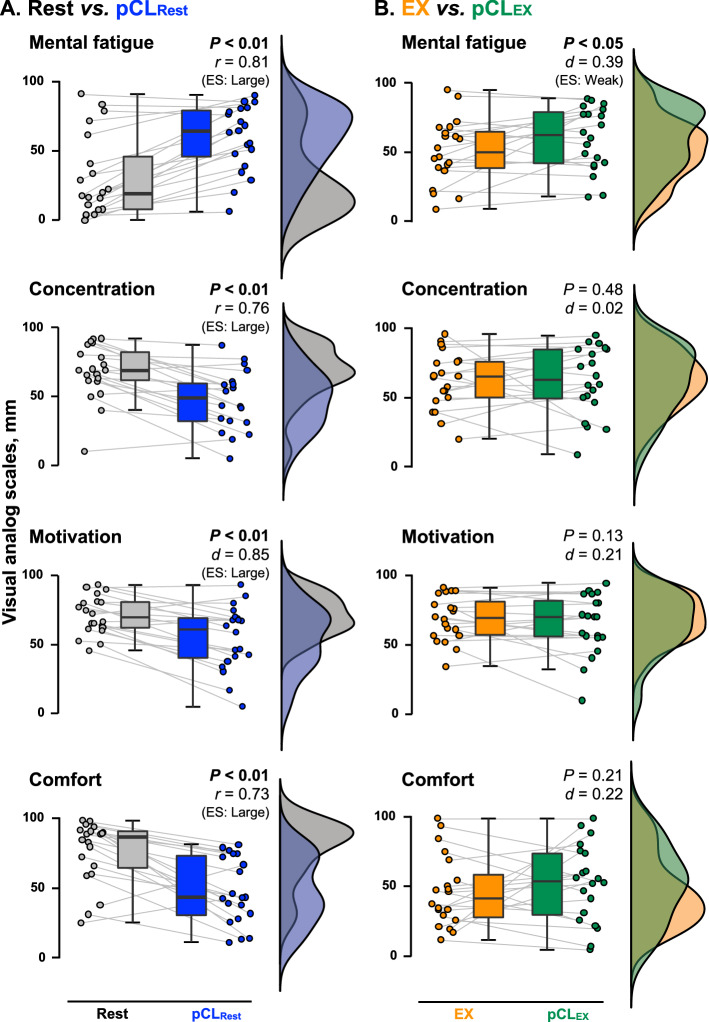
The psychological parameters. (**A**) State mental fatigue, concentration, motivation, and comfort immediately after a 50-min resting period with (pCL_Rest_; blue) and without (Rest; gray) prolonged cognitive load (pCL). (**B**) Those psychological parameters immediately after a 50-min exercise period with (pCL_EX_; green) and without (EX; orange) pCL. Raincloud plots showing the distribution of each psychological parameters assessing visual analog scale (VAS). Circle plots represent individual data, and box-and-whisker plots are median (IQR and max/min). *ES;* Effect size.

**Figure 2:**
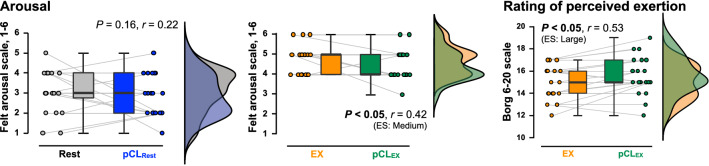
The arousal and rating of perceived exertion (RPE). Raincloud plots showing the distribution of arousal and RPE. Circle plots represent individual data, and box-and-whisker plots are median (IQR and max/min).

The pCL increased mental fatigue during both rest (*P* < 0.01, *r* = 0.81; Fig. 1A) and exercise conditions (*P* < 0.05, *d* = 0.39; Fig. 1B). Lower concentration (*P* < 0.01, *r* = 0.76), motivation (*P* < 0.01, *d* = 0.85) and comfort (*P* < 0.01, *r* = 0.73) were observed in pCL_Rest_ compared to Rest, but not between exercise conditions (concentration *P* = 0.48, *d* = 0.01, motivation *P* = 0.13, *d* = 0.21, and comfort *P* = 0.21, *d* = 0.22). In contrast, arousal remained unaltered following pCL_Rest_ (*P* = 0.16, *r* = 0.22), whereas a lower arousal level was observed during pCL_EX_ compared to EX condition (*P* < 0.05, *r* = 0.42; Fig. 2). Moreover, although the heart rate (HR) response to exercise was similar between EX (156 ± 11 bpm) and pCL_EX_ (157 ± 15 bpm) conditions (*P* = 0.87, *d* = 0.03), a rate of perceived exertion (RPE) was increased by the pCL (*P* < 0.05, *r* = 0.53; Fig. 2).

### Executive function

**Figure 3:**
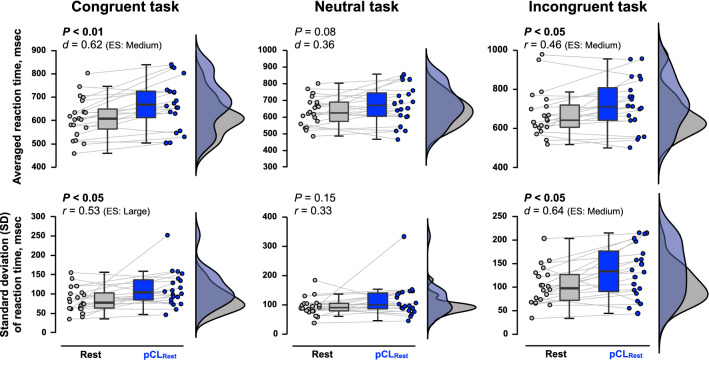
The reaction time (RT) of colour-word Stroop task (CWST) during the last 5 min of Rest (gray) and pCL_Rest_ (blue) conditions. Raincloud plots showing the distribution of RT data. Circle plots represent individual data, and box-and-whisker plots are median (IQR and max/min). The upper illustrations are the averaged data during 5-min collection, while the lower illustrations are the SD during 5-min data collection.

**Figure 4:**
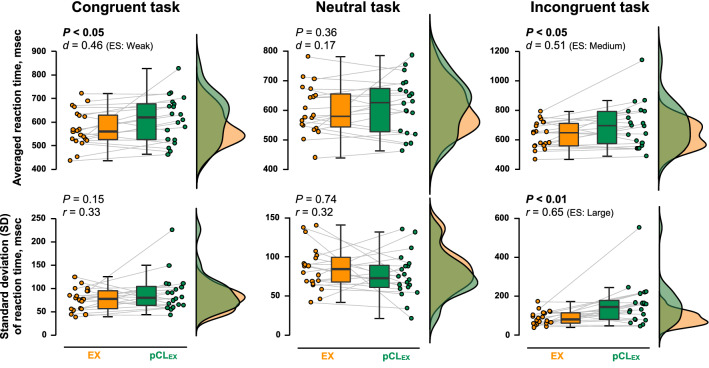
RT of CWST during the last 5 min of EX (orange) and pCL_EX_ (green) conditions. Raincloud plots showing the distribution of RT data. Circle plots represent individual data, and box-and-whisker plots are median (IQR and max/min). The left side illustrations are the averaged data during 5-min collection, while the lower illustrations are the SD during 5-min data collection.

**Figure 5:**
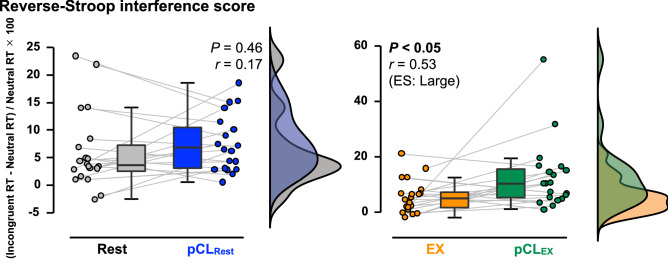
The reverse-Stroop interference score during the last 5 min of interventions. Raincloud plots showing the distribution of reverse-Stroop interference score. Circle plots represent individual data, and box-and-whisker plots are median (IQR and max/min).

**Figure 6:**
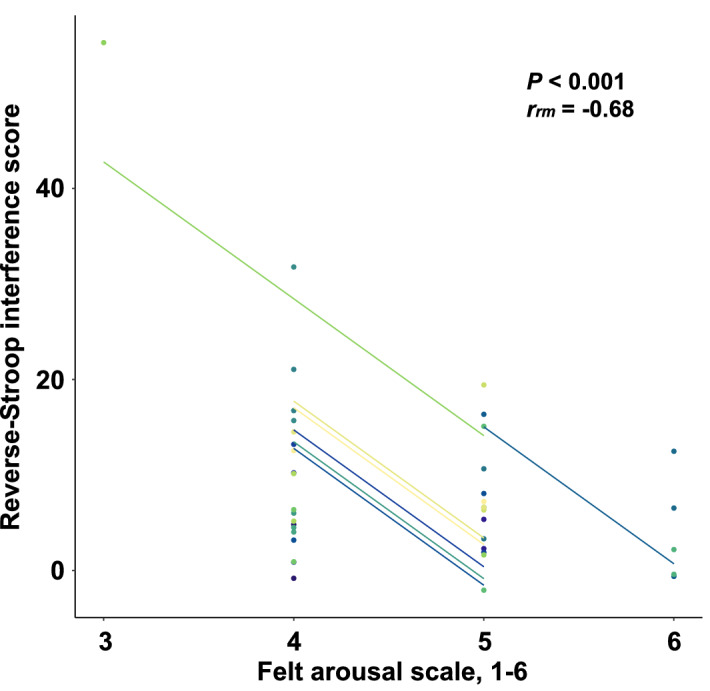
Repeated measures correlations (rmcorr) between the reverse-Stroop interference score and arousal in response to EX and pCL_EX_ conditions. Circle plots represent individual data, and lines show the rmcorr coefficient (*r*_*rm*_) fit for the participants. Same colour represent data from the same participants.

The number of errors for all CWST were not different between conditions (Table 1). When comparing Rest and pCL_Rest_ (Fig. 3), pCL caused a slower averaged RT of both congruent (*P* < 0.01, *d* = 0.62) and incongruent tasks (*P* < 0.05, *r* = 0.46). In addition, the SD of RT (*i.e.*, intraindividual variability) for both congruent (*P* < 0.05, *r* = 0.53) and incongruent tasks (*P* < 0.01, *d* = 0.64) were also increased by the pCL_Rest_. Similarly, compared with EX, the slowed RT (congruent task-averaged RT *P* < 0.05, *d* = 0.46; incongruent task-averaged RT *P* < 0.05, *d* = 0.51) and large SD of RT (incongruent task-SD of RT *P* < 0.01, *r* = 0.65) were observed in pCL_EX_, except for the SD of RT for congruent task (*P* = 0.15, *r* = 0.33; Fig. 4). The reverse-Stroop interference score was not affected by pCL_Rest_ (*P* = 0.46, *r* = 0.17) but was increased (*i.e.*, declined EF) by the pCL_EX_ compared with EX condition (*P* < 0.05, *r* = 0.53; Fig. 5). The increased reverse-Stroop interference score was associated with the decreased arousal (*P* < 0.001, *r*_*rm*_ = -0.68; Fig. 6).

**Table 1:** The number of errors for each task of colour-word Stroop task.

*Rest trials*	Rest	pCL_Rest_	*P*-values
Congruent task, *n*	0 (0–0)	0 (0–0)	0.26
Neutral task, *n*	1 (0–1)	0 (0–1)	0.73
Incongruent task, *n*	0 (0–1)	0 (0–1)	0.51

*Exercise trials*	EX	pCL_EX_	*P*-values
Congruent task, *n*	0 (0–1)	0 (0–1)	0.53
Neutral task, *n*	0 (0–1)	0 (0–1)	0.48
Incongruent task, *n*	0 (0–1)	0 (0–1)	0.69

Values are median (IQR). *pCL;* prolonged cognitive load, *EX;* Exercise.

## Discussion

The present study examined the impact of continuous pCL on RT and EF during acute moderate-intensity aerobic exercise. In line with our hypothesis, a slower RT and diminished EF was apparent during exercise with pCL, along with the increased RPE and mental fatigue. Namely, pCL enhanced cognitive fatigue even during aerobic exercise.

To avoid the speed-accuracy trade-off, all participants paid attention to prevent mistakes in CWST throughout all conditions (see Methods). Accordingly, the error of CWST was almost 0 in all conditions (see Table 1). Thus, we focused on the interpretation of changes in RT of CWST in the present study. Moreover, a previous study demonstrated that prolonged CWST (> 30 min) causes a slower averaged RT and large intraindividual RT variability^4^. At rest, we also confirmed that the 50-min CWST caused the same response. In addition, mood was impaired by the 50-min CWST at rest. Thus, our 50-min CWST is capable of inducing cognitive fatigue.

Acute moderate-intensity aerobic exercise increases state mental fatigue^13,14^. In addition, we have revealed for the first time that exercise-induced mental fatigue and perceived effort is further enhanced by pCL. These observations indicate that the subjective marker of internal load (*i.e.*, RPE) was increased by the continuous cognitive effort, despite similar absolute (*i.e.*, W) and physiological (*i.e.*, HR) intensities. The increased RPE may be associated with the hyperventilation-induced suppression of cerebral blood flow (CBF) in response to aerobic exercise^21^ or activation within the posterior cingulate cortex and precuneus, as demonstrated using fMRI^22^. Given that the dorsolateral prefrontal cortex is activated during EF performance^23^ despite de-activation during aerobic exercise^24^, it is possible that the brain during a 50-min dual-task with motor and cognitive demands may need higher energy requirements (*i.e.*, activate both anterior and posterior areas) whereas global CBF as energy supply may be decreased by moderate-intensity aerobic exercise-induced hyperventilation^12^. Hence, compared with exercise only, mental fatigue and RPE are further increased by the combined effort of exercise and pCL possibly due to cerebral energy deficiency.

In relation to mental fatigue and RPE, a slower averaged RT and large intraindividual RT variability were observed during aerobic exercise with pCL in the same way as the rest conditions. These observations indicated that the maintained cognitive effort causes a greater delayed RT and the unstable RT during the last 5-min of 50-min exercise. Incidentally, pCL did not alter the SD of RT for the congruent task during exercise, implying that the pCL-induced intraindividual simple RT variability (*i.e.*, stability of performance for easy task) may be masked by the effect of exercise. Importantly, EF (*i.e.*, interference) during exercise was diminished by the 50-min pCL (*i.e.*, dual-task), despite no effect of the single-task of pCL (*i.e.*, rest). It is likely that the delayed decision-making speed during the prolonged dual-task may be partly evoked by the impaired cognitive process of EF. Given that the speed of decision-making and EF may contribute to superior sports performance^1,25^, cognitive central fatigue may adversely impact sports performance.

The physiological mechanism(s) of this finding remains unknown. However, it may be linked to psychological arousal. Psychological arousal is associated with the exercise-induced improvements in EF^26^. Correspondingly, a lower level of psychological arousal may be associated with the pCL-induced impairment in EF during exercise (see Fig. 6). Therefore, the results of the present study may be explained by biomarkers of physiological arousal. Alternatively, we previously reported that dynamic cerebral autoregulation (dCA) may be maintained/improved by exercise/diet stress^27,28^ which is capable of facilitating speed of decision-making^29,30^. The dCA acts to maintain relatively constant CBF against rapid fluctuations in perfusion pressure for brain homeostasis^31^, but Ogoh et al.^32^ demonstrated that a 5 min cognitive performance-induced cerebrovasodilation (*i.e.*, brain activation) impaired dCA. Future studies should examine whether pCL impairs the dynamic CBF regulation at rest and during exercise, thereby contributing to cognitive decline.

### Implications for brain health

EF has an important role in brain health and deteriorations have been observed in neurological conditions such as dementia^33^. It is widely known that regular and chronic exercise training improves EF^34^. For instance, evidence exists suggesting that a 3–12 month dance program as a simultaneous motor-cognitive exercise with an ‘incorporated’ cognitive task^35^ enhances EF^36^. Interestingly, a 3-month intervention of 40-min dual-task “Cognicise (Cognition + Exercise of low-intensity)” may improve cognitive function for the elderly who have a cognitive impairment although to what extent aerobic exercise alone could have improved cognition in this previous study was not investigated^37^.

The exercise training-induced improvements in cognition may be predicted by the acute effects of exercise^38^. Of note, the effect size of a single bout of moderate-intensity exercise on EF improvement is greater than low-intensity exercise^13^. Nevertheless, the present study implies that the inhibitory control in EF may be diminished when cognitive performance is maintained during moderate-intensity aerobic exercise prescription, indicating the strong negative effect of cognitive fatigue due to simultaneous motor-cognitive exercise with an ‘additional’ cognitive task^35^. Moreover, Kamijo and Abe^39^ demonstrated that working memory in EF is improved after 20-min moderate-intensity aerobic exercise but this aftereffect is cancelled by a 20-min additional cognitive performance during moderate-intensity aerobic exercise. In addition to EF, pCL increased RPE in the present study. The higher level of RPE may be associated with a lower level of psychological self-efficacy^40^, which predicts lower exercise adherence^41^. In light of these findings, there is, on the one hand, evidence that chronic physical exercise with an additional or incorporated cognitive task (*e.g.*, dancing) can improve cognitive performance, whereas, on the other hand, our findings imply that acute physical exercises with an additional cognitive task can cause cognitive fatigue which, in turn, negatively influence cognitive performance with respect to shorter time frames. To optimize the prescription of physical exercise for cognitive health, future research on physical exercise with additional or incorporated cognitive tasks is needed.

### Perspective

In football, an increase in goal-scoring rate is observed in the latter stages of the match along with fatigue^42^, which may partly be due to the impairment in cognitive perception^3^. However, Wiśnik et al.^43^ reported that the RT of cognitive task is shortened during treadmill exercise of changing intensity simulating a sport game. To the best of our knowledge, previous research could not detect why the cognitive-perceptive skills may often be poorer in the latter stage of the match. For example, Wang et al.^44^ demonstrated that EF is diminished during exhaustive high-intensity continuous exercise (*e.g.*, 80%VO_2_ peak for 40-min), but the continuous exercise may not reflect the sporting performance and training situation, except for marathon etc. The present findings provide an alternative explanation for physical fatigue as to why sports performance may be impaired towards the end of the game^3^. Although the cognitive-perceptive skills in sports may be altered by confounding factors such as contacts^45–47^, we proposed a new experimental design, which can examine the impact on cognitive fatigue during prolonged aerobic exercise. Future studies are necessary to reveal training/nutritional strategies that may improve EF and psychological variables during this dual-task, which in turn could mitigate the negative effects and subsequently improve sports performance.

### Conclusion

A 50-min cognitive task impaired RT not only at rest, but also during moderate-intensity aerobic exercise. In addition, EF during aerobic exercise was diminished when the cognitive task was continuously performed. These findings provide empirical evidence that cognitive fatigue appears even whilst aerobic exercising and impairs cognitive-perceptive skills during aerobic exercise.

## Methods

### Ethics and participants

All procedures were approved by the Ethics Committee of Ritsumeikan University (BKC-2020–085) and conformed to the *Declaration of Helsinki*, except for registration in a database. A priori sample size calculation (G*Power) suggested that assuming the variation in reaction time following the completion of a prolonged modified-CWST^6^, a sample size of 21 would provide a statistical power of 80% at an α level of 0.05. Accordingly, twenty-one healthy males participated in the study after providing written informed consent. All participants were free of neurologic, cardiovascular or pulmonary disorders, were not taking any medication, and were non-smokers. Participants were instructed to avoid alcohol, caffeine intake and strenuous physical activity in the 24-h preceding each experimental visit. Each participant was also asked to abstain from food for 12-h before each experiment. Experiments were performed at 22–24 ℃.

#### Study design (Fig. 7)

**Figure 7:**
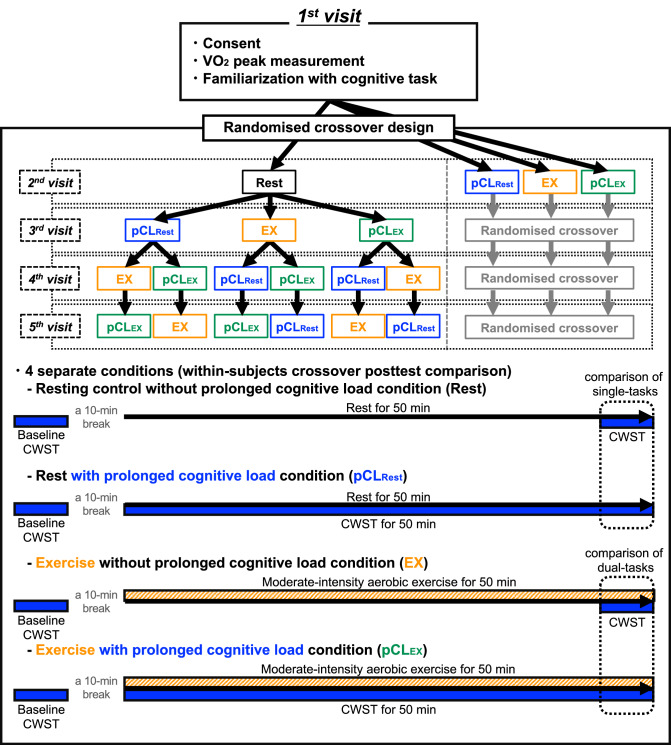
Schematic representation of the experimental design.

Participants initially completed a preliminary session where they were familirised with the cognitive assessment (*i.e.*, CWST) and their cardiorespiratory fitness was determined. Thereafter, 4 separate conditions were completed on separate days in a randomized, counterbalanced order: Rest, pCL_Rest_, EX, and pCL_EX_. Participants attended the experimental days between 08:00–10:00 am and each visit was separated by a minimum of 72-h.

#### Experimental conditions (Fig. 7)

During all conditions, participants performed CWST after resting for 10-min to determine the speed and accuracy of the decision-making and EF at baseline. HR and psychological state were also measured prior to each intervention. After confirming a slight day-to-day difference at baseline (Supplementary Table 1), we performed the experiment using within-subjects crossover posttest comparison, the most robust study design to investigate the acute effects of physical exercise on cognitive performance^15^. In the Rest condition, the participants performed 5-min CWST after sitting at rest on the bike for 45-min. In the EX condition, the participants performed moderate-intensity cycling exercise for 50-min, and CWST was carried out during the final 5-min. In the pCL_Rest_ condition, the participants continuously performed CWST for 50-min in a sitting position on the bike. In the pCL_EX_ condition, the participants continuously performed both CWST and moderate-intensity cycling exercise for 50-min. In both pCL_Rest_ and pCL_EX_ conditions, the speed and accuracy of the decision-making, including EF, was determined from the score of CWST at the final 5-min of such interventions.

### Experimental procedures and measurements

#### Cardiorespiratory fitness

VO_2_ peak was determined on a bike (95C Inspire Upright Lifecycle, Life Fitness Japan, Tokyo, Japan). The exercise test began at a power of 60 W for 1 min. Thereafter, the workload was increased by 15 W/min until the participants could not maintain a cadence of 60 rpm (task failure of a pedaling rate of at least 55 rpm over 5 s despite maximal effort)^27,48^. Throughout the test, expired gas fractions were measured continuously using online breath-by-breath gas analysis (AE-310S; Minato Medical Science, Osaka, Japan). VO_2_ peak was determined as the highest 30-s value attained prior to exhaustion^13,14,30,49^ and used to calculate moderate-intensity exercise for the relevant experimental conditions (60% VO_2_ peak; 151 ± 18 W).

#### Heart rate

HR was monitored at rest and during exercise using telemetry (RS400 and H10; Polar Electro, Kempele, Finland).

#### Psychological parameters

The visual analog scale (VAS) included questions of 4 psychologic types that assessed mental fatigue, ability to concentrate for CWST, motivation for CWST, and comfort. Each VAS was labeled from 0 (not at all) to 100 mm (extremely). The participants drew lines to indicate their responses^13,14,30,48^. Moreover, the Felt Arousal Scale (FAS) assessed arousal level, ranging from 1 (low arousal) to 6 (high arousal) and the participants were asked to report how they felt after performing the CWST. A high arousal level represents excitement, and a low arousal level represents relaxation^50^.

Immediately after exercise, participants were asked to provide RPE using the Borg 6–20 scale, ranging from 6 (no exertion) to 20 (maximal exertion)^51^ to estimate the effort expended during exercise.

#### Reaction time and accuracy of executive function task

Evaluation of the speed and accuracy of the decision-making and EF was completed using CWST^52^, a paradigm for investigating aspects of cognitive performance. This specifically provides selective attention to specific information and inhibits prepotent responses during decision-making tasks involving stimuli and responses^53^. The CWST was adopted in an event-related design and both 5-min and 50-min CWST were programmed in SuperLab (Cedrus, San Pedro, CA, United States). The words stimuli were 4-colour names; red, blue, yellow, and green in Japanese characters. The participants performed 3 types of CWST: 1, the congruent task as a facilitated easy task displaying the colour names presented in the same-coloured text; 2, the neutral task as the control displaying the color names presented in black text; and 3, the incongruent task as an interference task displaying the color names in a different coloured text. The words for each type of task were presented for 1500 ms following a 500 ms fixation cross, in a counter-balanced random order and random interval (1, 3, and 5 s) to avoid prediction of the timing of the subsequent task^4^; in other words, 20 congruent, 20 neutral and 20 incongruent stimuli were presented per 5-min CWST. A colour-labeled keyboard (RB-540, Cedrus, San Pedro, CA, United States) was prepared and the participants were asked to press the colour-labeled key that corresponded to the text meaning of the stimulus word. The following instruction was given to the subjects; “Please do not make a mistake” and “You must perform the task as accurately and quickly as possible”. The averaged and SD of RT, and the number of errors were assessed using 5-min data. To evaluate EF, reverse-Stroop interference scores were calculated as the averaged RT as [(RT of incongruent task−RT of neutral task) / RT of neutral task)] × 100 because a higher and stable reverse-Stroop interference scores can be observed when CWST is performed using manual response^54^. In a preliminary session, CWST was performed during cycling exercise for approximately 10-min (*i.e.*, a dual-task) until the participants obtained consistent scores to avoid learning effect.

### Statistical analysis

If distribution of normality was confirmed using Shapiro Wilk tests, table/supplemental table data are expressed as means ± SD. Where table/supplemental table data were not normally distributed, they are expressed as median (IQR). In terms of the figures, individual, box-and-whisker, and raincloud plots were created by using JASP software (version 0.16.4, University of Amsterdam, Netherlands)^55^. Baseline data were analyzed using two-way (pCL × EX) repeated-measures analysis of variance after normal data distribution was confirmed, whereas the Friedman test was used to analyze the baseline data if normal data distribution was not confirmed (IBM SPSS Statistics version 27, Chicago, IL, United States). In addition, whereas we did not directly compare the data between the rest and exercise conditions because of the influence of different test conditions due to the dual-task (*e.g.*, the percentage of effort to CWST test, gaze destabilization, and sweating, etc.), a paired *t*-test (for normal data distribution) and the Wilcoxon signed-rank test (for non-normal data distribution) were used to compare the data between Rest and pCL_Rest_, and between EX and pCL_EX_, respectively (IBM SPSS Statistics version 27, Chicago, IL, United States). The statistical significance level was defined at *P* < 0.05. Moreover, Cohen’s *d* effect size using the means and pooled SD were calculated, along with the 95% confidence interval to determine the magnitude of differences for normal data distribution. For non-normal data distribution, the effect size was estimated as *r* using *Z*-score for the Wilcoxon signed-rank test. The strength of effect size of Cohen’s *d* was interpreted as weak (0.20 ≤ *d* < 0.50), medium (0.50 ≤ *d* < 0.80), and large (0.80 ≤ *d*), while the strength of effect size of *r* was interpreted as weak (0.10 ≤ *r* < 0.30), medium (0.30 ≤ *r* < 0.50), and large (0.50 ≤ *r*)^56^. Finally, we performed repeated measures correlation (rmcorr) between the reverse-Stroop interference and arousal^57^.

## Supplementary Information

Supplementary Information.

## Data Availability

The data that support the findings of this study are available from the corresponding author, H.Tsukamoto, upon reasonable request.
