# Supplementary material for: Executive function during exercise is diminished by prolonged cognitive effort in men
Source: Sci Rep. 2022 Dec 27;12:22408. doi: 10.1038/s41598-022-26788-6 (PMC9794810; doi:10.1038/s41598-022-26788-6)
Supplement: Supplementary file 1 — Supplementary Information. [file 41598_2022_26788_MOESM1_ESM.docx]

Supplemental Table 1. Baseline states

|  |  |  | Rest | pCL_Rest_ | EX | pCL_EX_ | *P*-values | | |
| --- | --- | --- | --- | --- | --- | --- | --- | --- | --- |
|  |  |  |  |  |  |  | pCL | EX | Interaction or |
|  |  |  |  |  |  |  |  |  | Overall |
| Heart rate, bpm | | | 72 ± 11 | 70 ± 10 | 72 ± 11 | 70 ± 10 | 0.21 | 0.88 | 0.96 |
| *Psychological states* | | |  |  |  |  |  |  |  |
|  | Visual analog scales, 0-100 mm | |  |  |  |  |  |  |  |
|  |  | Mental fatigue, mm | 17 (3-37) | 16 (6-31) | 9 (4-33) | 13 (5-26) | N/A | N/A | 0.50 |
|  |  | Ability to concentrate, mm | 70 ± 19 | 67 ± 23 | 71 ± 20 | 68 ± 22 | 0.32 | 0.73 | 0.91 |
|  |  | Motivation, mm | 77 (60-90) | 79 (56-89) | 83 (57-92) | 78 (63-88) | N/A | N/A | 0.85 |
|  |  | Comfort, mm | 89 (75-95) | 87 (57-97) | 89 (67-94) | 88 (66-95) | N/A | N/A | 0.77 |
|  | Felt arousal scale | |  |  |  |  |  |  |  |
|  |  | Arousal, 1-6 | 3 (2-3) | 3 (2-3) | 3 (2-3) | 3 (2-3) | N/A | N/A | 0.66 |
| *Colour-word Stroop test* | | |  |  |  |  |  |  |  |
|  | Congruent task | |  |  |  |  |  |  |  |
|  |  | Averaged reaction time, ms | 622 ± 94 | 617 ± 95 | 604 ± 73 | 637 ± 103 | 0.31 | 0.97 | 0.16 |
|  |  | SD of reaction time, ms | 93 ± 32 | 95 ± 35 | 91 ± 32 | 116 ± 47 | 0.06 | 0.27 | 0.09 |
|  |  | Error, *n* | 0 (0-0) | 0 (0-1) | 0 (0-0) | 0 (0-1) | N/A | N/A | 0.17 |
|  | Neutral task | |  |  |  |  |  |  |  |
|  |  | Averaged reaction time, ms | 653 ± 94 | 644 ± 98 | 630 ± 75 | 658 ± 102 | 0.47 | 0.69 | 0.15 |
|  |  | SD of reaction time, ms | 94 ± 28 | 110 ± 41 | 104 ± 33 | 100 ± 32 | 0.31 | 0.97 | 0.08 |
|  |  | Error, *n* | 0 (0-1) | 0 (0-1) | 0 (0-1) | 0 (0-0) | N/A | N/A | 0.33 |
|  | Incongruent task | |  |  |  |  |  |  |  |
|  |  | Averaged reaction time, ms | 689 (614-761) | 704 (590-802) | 689 (600-756) | 679 (597-806) | N/A | N/A | 0.68 |
|  |  | SD of reaction time, ms | 109 (86-150) | 107 (93-182) | 101 (79-182) | 129 (83-162) | N/A | N/A | 0.85 |
|  |  | Error, *n* | 0 (0-2) | 0 (0-1) | 0 (0-1) | 0 (0-1) | N/A | N/A | 0.67 |
|  | Interference score, % | | 6.7 (3.0-11.4) | 8.5 (7.3-16.4) | 5.6 (2.7-10.3) | 5.8 (4.5-11.2) | N/A | N/A | 0.17 |

Values are mean ± SD or median (IQR). pCL, prolonged cognitive load; EX, exercise.
